# An ApiAP2 member regulates expression of clonally variant genes of the human malaria parasite *Plasmodium falciparum*

**DOI:** 10.1038/s41598-017-12578-y

**Published:** 2017-10-25

**Authors:** Rafael M. Martins, Cameron R. Macpherson, Aurélie Claes, Christine Scheidig-Benatar, Hiroshi Sakamoto, Xue Yan Yam, Peter Preiser, Suchi Goel, Mats Wahlgren, Odile Sismeiro, Jean-Yves Coppée, Artur Scherf

**Affiliations:** 1 Unité Biologie des Interactions Hôte-Parasite, Institut Pasteur, Paris, 75015 France; 2 CNRS, ERL 9195, Paris, 75015 France; 3 INSERM, Unit U1201, Paris, 75015 France; 4 School of Biological Sciences, Nanyang Technological University, Singapore, 637551 Singapore; 5 MTC, Nobels väg 16, KI Solna Campus Karolinska Institutet, Box 280, SE-171 77 Stockholm, Sweden; 6 Plateforme 2, Transcriptome et Epigenome, Institut Pasteur, Paris, 75015 France; 7 CNRS 5290/IRD 224/University of Montpellier (“MiVEGEC”), Montpellier, France; 8 Institute of Science Education and Research (IISER), Tirupati Rami Reddy Nagar, 517507 Mangalam, Tirupati Andhra Pradhesh India

## Abstract

Variegated surface antigen expression is key to chronic infection and pathogenesis of the human malaria parasite *Plasmodium falciparum*. This protozoan parasite expresses distinct surface molecules that are encoded by clonally variant gene families such as *var*, *rif* and *stevor*. The molecular mechanisms governing activation of individual members remain ill-defined. To investigate the molecular events of the initial transcriptional activation process we focused on a member of the apicomplexan ApiAP2 transcription factor family predicted to bind to the 5′ upstream regions of the *var* gene family, AP2-exp (PF3D7_1466400). Viable AP2-exp mutant parasites rely on expressing no less than a short truncated protein including the N-terminal AP2 DNA-binding domain. RNA-seq analysis in mutant parasites revealed transcriptional changes in a subset of exported proteins encoded by clonally variant gene families. Upregulation of RIFINs and STEVORs was validated at the protein levels. In addition, morphological alterations were observed on the surface of the host cells infected by the mutants. This work points to a complex regulatory network of clonally variant gene families in which transcription of a subset of members is regulated by the same transcription factor. In addition, we highlight the importance of the non-DNA binding AP2 domain in functional gene regulation.

## Introduction

Worldwide, 3.3 billion people are at risk of malaria, which in 2013 afflicted 198 million people, causing 584,000 deaths^1^. The deadliest form of the disease is caused by the apicomplexan parasite *Plasmodium falciparum*. Pathogenesis and severe malaria are linked to parasite adherence to endothelial cells leading to disruption of blood flow in small capillaries in critical organs. At the molecular level this phenomenon is mediated by parasite proteins expressed on the surface of infected red blood cells. In particular, the PfEMP1 (*P. falciparum* erythrocyte membrane protein 1) proteins, encoded by the 60-members *P. falciparum var* gene family, associated with severe malaria^2,3^. Expression of *var* genes is mutually exclusive, i.e. only one single *va*r gene is expressed at a time in a single cell and switching among the different members of the family allows *P. falciparum* to evade the host immune response, a process called antigenic variation^3,4^. *P. falciparum* also expresses other multigene virulence antigens that are exported to the surface of the red blood cell, the RIFINs, STEVORs, PfMC-2TMs (*P. falciparum* Maurer’s clefts-two transmembrane) and SURFINS, which are also clonally variant^5,6^. Growing evidence suggests these proteins play roles in key biological processes as diverse as invasion, rosetting, membrane rigidity and trafficking^6–12^. In contrast to *var* genes, which are also present in *P. reichenowi* ^13^, a subset of members of these gene families is expressed in a single parasite and they have orthologues in all *Plasmodium* species investigated so far^7,14,15^.

Although epigenetic mechanisms contribute to clonally variant expression of these gene families, specific factors that initiate the transcription process remain elusive^16,17^. In 2005, with the discovery of the Apetala2 (AP2) family of transcriptional regulators in malaria parasites, formerly found exclusively in plants and algae, research on specific transcriptional mechanisms in *Plasmodium* and other apicomplexan parasites gained considerable momentum^18^. Twenty-seven distinct AP2 members are predicted bioinformatically in *P. falciparum* and the DNA motifs preferentially bound by recombinant AP2 domains of most of its members have been described^19^. In plants, these proteins are involved in different developmental processes and stress responses and the same protein can both activate and repress gene transcription^20–23^.

Initial work performed in *P. berghei* demonstrated the key role of AP2 members in regulating the development of sporozoites, ookinetes, liver and sexual stages, namely AP2-Sp, AP2-O, AP2-L and AP2-G^24–27^. Genome-wide occupancy studies have shown AP2-O to bind the promoters of most genes deregulated in the knockout parasite^28^ and that AP2-G2 targets about 1500 genes mostly required for asexual replication, repressing them before commitment to sexual differentiation^29^. More recently, a knockout screen of ApiAP2 genes in *P. berghei* was published, unraveling the critical functions of several ApiAP2s in different stages of the parasite life-cycle^30^.

In *P. falciparum* three AP2s have been functionally characterized at the cellular level. PfSIP2 (*Plasmodium falciparum* SPE2-interacting protein) binds preferentially to telomeric-associated repetitive elements (TAREs) 2/3 regions and was suggested to play a role in heterochromatin formation^31^. Another AP2 is part of a multiprotein complex bound to upsC *var* introns with a suggested role in tethering *var* genes to the nuclear periphery^32,33^. Recently, PfAP2-G was revealed as a master regulator of sexual commitment^34^. Campbell *et al*.^19^ uncovered a list of putative DNA-binding motifs for *P. falciparum* ApiAP2 members by using recombinant proteins corresponding to the predicted DNA-binding domains^19^. The motifs for three AP2s: PF3D7_1466400 ([geneDB nomenclature] herein called AP2-exp), PF3D7_1143100 and PF3D7_0604100 (PfSIP2) were reported to be enriched in *var* gene promoters. In this work, we sought to define the role of AP2-exp in virulence genes regulation in *P. falciparum*. The orthologue of this protein in the murine malaria parasite *P. berghei*, AP2-Sp, has been shown to be expressed exclusively in the sporozoite stage^24^ but recently, in a knockout screen of *P. berghei* AP2s, the authors observed a slight phenotype in blood stages, accompanied by transcriptional deregulation of 265 genes^30^. Using an antibody specific to the AP2 domain, we found the protein to be expressed in the nuclear fraction of late blood stages of *P. falciparum*. By means of a genetic approach, the complete deletion of *AP2-exp* was unsuccessful, suggesting the protein plays essential roles during the intraerythrocytic cycle (IEC). Nevertheless, we generated mutants overexpressing a truncated N-terminal version of the gene including the AP2 DNA-binding domain. Although mutually-exclusive *var* gene expression in ring stages 14 hours post-invasion (hpi) was unaltered in the mutants, upregulation of members of other clonally variant gene families and putative exported proteins was observed by deep-sequencing of the transcriptome of mutant parasites in late stages (30 hpi). Upregulation of RIFINs and STEVORs was confirmed by western blot analysis and the knobs, structures associated to infected red blood cell cytoadhesion, were morphologically altered. Our work suggests that the non-DNA-binding AP2-exp region is a critical factor that contributes to the regulation of a subset of virulence genes in *P. falciparum*.

## Results

### Disruption of the *AP2-exp* gene produces viable blood stage parasites

**Figure 1:**
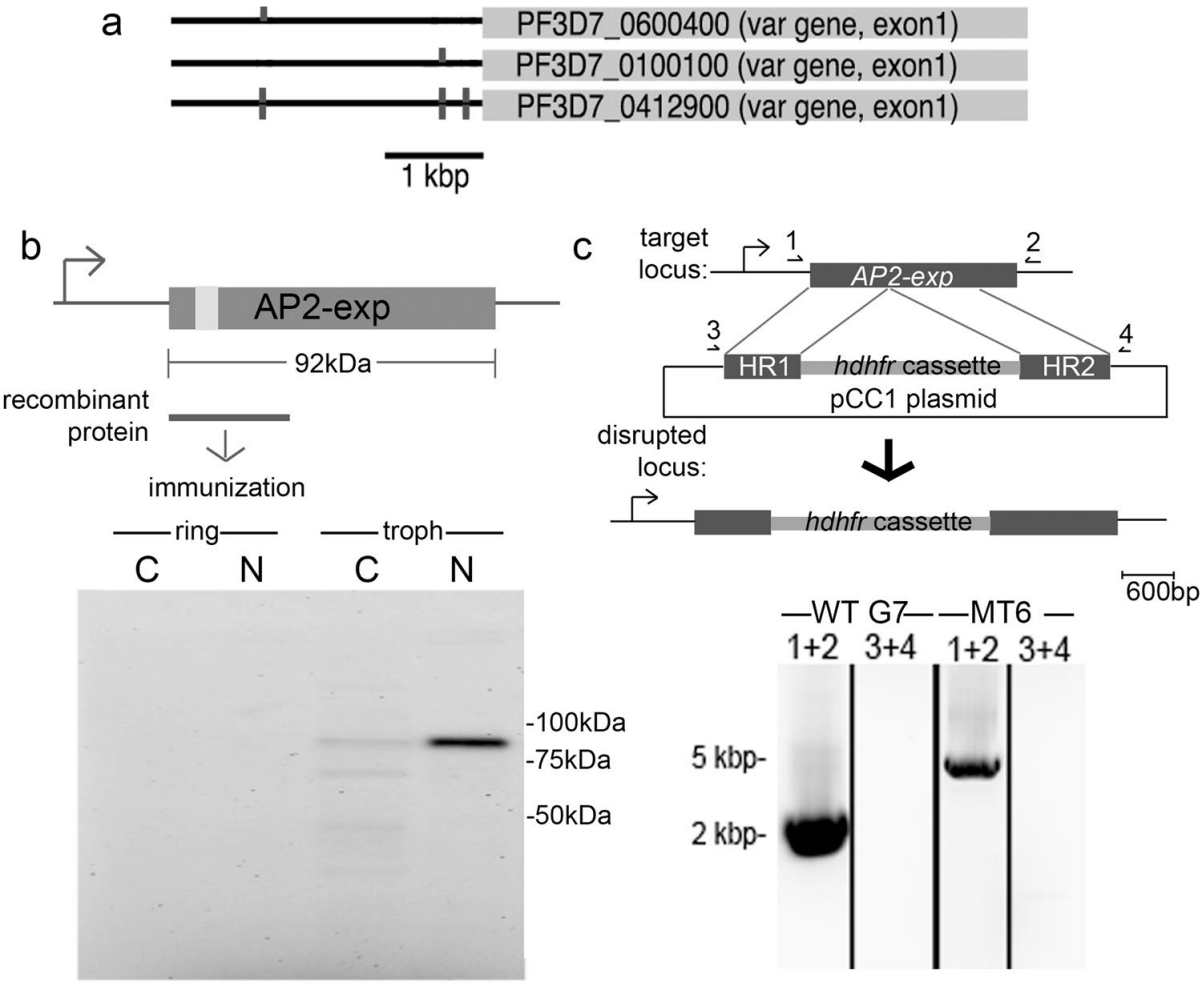
Strategies to investigate AP2-exp. (**a**) Predicted AP2-exp binding sites (vertical boxes) in 5′ UTRs of upsA, upsB and upsC *var* genes. (**b**) Top, AP2 domain (light gray box) of AP2-exp. Line shows the region used for obtention of specific antibodies. Bottom, western blot using anti-AP2-exp antibodies on cytoplasmic (C) and nuclear (N) extracts of early (ring) and late (troph) stages of 3D7 parasites. (**c**) Strategy used to disrupt the *AP2-exp* locus: the *hdhfr* cassette was inserted by double-crossover recombination. 1 and 2, 3 and 4 are oligonucleotides to detect recombination. Bellow, PCR on 3D7 wild-type (wt) and one mutant clone (mt 6). 5 kbp and 2 kbp molecular markers indicated on the left.

To shed light into the molecular process that initiates activation of clonally variant gene families in heterochromatin regions, we decided to investigate the role of an ApiAP2 member termed here AP2-exp (PF3D7_1466400) that was predicted to bind to promoter regions of *var* genes^19^. In Fig. 1a examples are shown of the localization of these motifs in different types (*upsA, upsB* and *upsC*) of *var* genes. In this family, only a single member is normally expressed, being part of the general parasite strategy to employ phenotypic variation for immune evasion^35,36^. *AP2-exp* is predicted to code for a 92 kDa protein containing a single AP2 domain at the N-terminal region (light gray box on Fig. 1b). We produced a recombinant glutathione-S transferase (GST)-fusion protein containing the AP2 domain to immunize rats and obtain specific anti-AP2-exp antibodies. The antibodies predominantly recognized a band corresponding to the predicted size of the protein in nuclear extracts of late stage parasites (Fig. 1b, bottom), supporting a possible role of AP2-exp in gene regulation in the trophozoite/schizont stages.

To investigate the biological role of this protein, we chose to disrupt the gene using the pCC1 vector carrying two homology boxes flanking the human dihydrofolate reductase *(dhfr)* selectable marker (conferring resistance to the antifolate WR99210)^37^. Double-crossover recombination events were detected by the increase in the size of the PCR-amplified band of the locus shown in Fig. 1c. By limiting dilution, clones were obtained with the disrupted AP2-exp gene. Two clones were used for this study termed MT6 and MT20. Other constructs, designed to integrate in the 5′ UTR of the gene to entirely delete it proved unsuccessful in our hands, i.e., integration events were never detected, suggesting that the product of the full-length gene might be essential to blood stage proliferation. Moreover, we also tried to tag the full-length protein using the *E. coli* dhfr destabilization domain^38^ but no recombinants could be obtained. This is in contrast to the orthologue ApiAP2 from the murine malarial parasite *P. berghei* (called AP2-Sp), in which the gene knockout shows little phenotype in this part of the parasite cycle, but blocks sporozoite development in the mosquito^24,30^.

### Mutant parasites overexpress a truncated N-terminal region of AP2-exp

**Figure 2:**
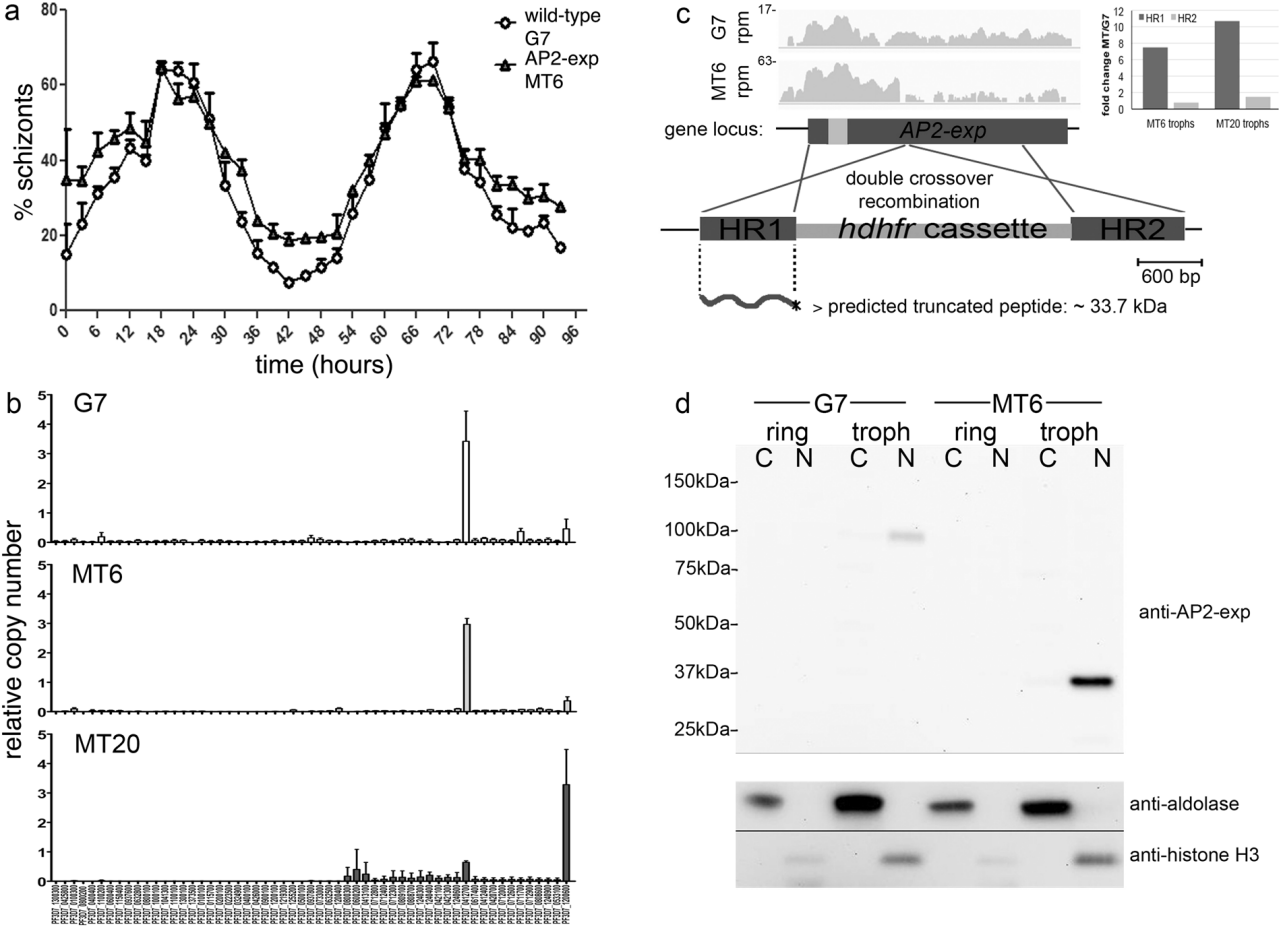
AP2-exp mutant parasites are viable and produce a short truncated protein. (**a**) One wild-type clone (circles, G7) and one mutant clone (triangles, MT6) were analysed by flow cytometry. (**b**) Single *var* genes expression profile on wild-type G7 clone and 2 AP2-exp mutants, MT6 and MT20. qRT-PCR was performed for individual *var* genes and relative copy number determined based on the seryl-tRNA synthetase gene. (**c**) Coverage of transcript reads (in reads per million, rpm) at the AP2-exp locus in the wild-type and mutant parasites at 30 hpi. Inset shows qRT-PCR confirming expression of homology region 1 in both MT6 and MT20. Below, translation of the disrupted locus in the mutants predicts a 33.6 kDa polypeptide. (**d**) Western blot of cytosolic and nuclear extracts from ring and trophozoite stages of G7 and MT6 parasites using anti-AP2-exp. Cytosol and nuclear controls were anti-Pfaldolase and anti-core human H3, respectively. Cropped bands are displayed and full-length membrane is in Supplementary Figure S5.

The cell cycle of the mutant parasites was analyzed by flow cytometry to detect potential defects in asexual blood stage development. Wild-type and AP2 mutant parasites had identical cell cycle timing (Fig. 2a). Since predicted binding sites for AP2-exp are enriched in upstream regions of *var* genes, we investigated transcript levels of the *var* gene family by quantitative real-time PCR (qRT-PCR), on cDNA prepared from 14 hours post-invasion (hpi) ring stages. The analysis of two mutant clones called MT6 and MT20 and 3D7 wild-type (G7 clone), demonstrated that only one major *var* gene was expressed, indicating that the mutually exclusive *var* gene expression was not disrupted in AP2-exp mutant parasites (Fig. 2b). Moreover, *var* gene expression decreased similarly in late stages of mutants and wild-type G7 (data not shown), ruling out a major role of this AP2 protein on stage-specific and monoallellic *var* expression. Though *var* genes are the main players in *P. falciparum* cytoadherence, other exported clonally variant families such as *rif*, *stevor* and *pfmc-2tm* are expressed at late asexual blood stages (trophozoite-schizont stage) at the erythrocyte membrane at the time AP2-exp is expressed. In order to get a genome-wide view on the role of AP2-exp on the transcriptional profile of *P. falciparum*, we performed RNA-seq analysis on trophozoites at 30 hpi. Libraries from poly-A-enriched RNA of 24–30 hpi trophozoites were sequenced in an Illumina platform, aligned using BWA MEM, and differential expression assessed by edgeR. When we looked at the *AP2-exp* locus, instead of an expected absence of reads due to the disruption of the gene and absence of a stable 3′ UTR, we observed more uniquely-mapped reads that could be aligned to the region corresponding to the first homology box designed for integration in the AP2-exp locus in the mutant line than in the wild-type. This was followed by an abrupt interruption in the coverage where the drug selection cassette *hdhfr* was integrated and a drastic reduction in the downstream part of the coding sequence (Fig. 2c), indicating that a transcript encompassing the AP2 DNA-binding domain was overexpressed in the AP2 mutant parasites. Indeed, by qRT-PCR, we found that the region corresponding to the homology box 1 was overexpressed 7.5 fold in trophozoites of MT6 and 10.7 fold in MT20 (inset in Fig. 2c). If translated, this transcript would generate a truncated polypeptide of around 33.7 kDa, containing the AP2 domain. Using the antibodies anti-AP2-exp in western blots (Fig. 2d), we did not detect the full-length protein in the nuclear and cytoplasmic fractions of the mutant parasites. However, a truncated protein, whose size corresponded to the prediction of the translation of the N-terminal region of AP2-exp was detected. Expression was still stage-specific, since we did not detect the protein in early, ring stages. Quantification of the signal of the protein bands suggested that the truncated protein was approximately 10-fold overexpressed relative to the wild-type protein.

### Mutant AP2-exp derepresses clonally variant gene families

**Figure 3:**
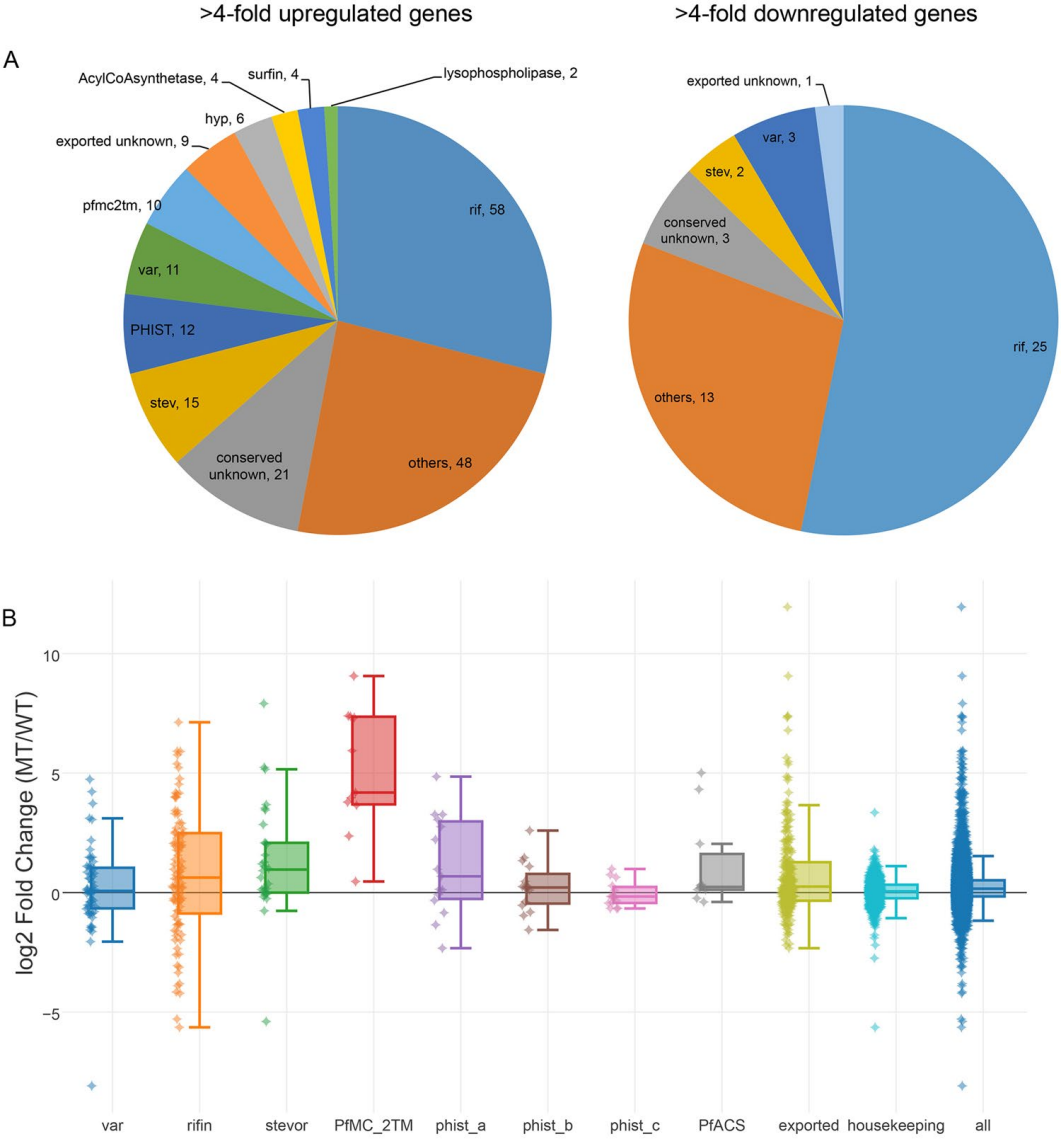
Differential gene expression in the mutant parasites. (**A**) Genes belonging to multigene families up- or down-regulated in the AP2-exp mutant parasites. Family names are indicated followed by the total number of differentially-expressed genes. (**B**) Box-and-whiskers plot of RNA-seq analysis of mutant parasites. Log2 fold-change in mutants versus wild-type is shown.

Analysing different biological replicates of the wild-type clone G7 and both mutant clones, MT6 and MT20, we observed 276 more than 4-fold differentially-expressed, mostly upregulated gene products (including annotated pseudogenes and truncated fragments): 222 upregulated and 54 downregulated (Fig. 3A and Supplementary Table S2). Discarding pseudogenes and truncated gene products, upregulated genes are 190 and downregulated, 47. They belong mostly to variant subtelomeric gene families that are clonally-expressed (Fig. 3 and Supplementary Figure S1), encompassing members of surface proteins encoded by *rif* and *stevor* gene families and the exported *PfMC-2TM* (2 transmembrane) gene family. Moreover, we detected deregulation of a few *var* genes, though at this time of the cycle, 30 hpi, var gene transcripts decrease, and a subgroup of the *phist* gene family (*phist-a*) showed significant derepression. With the exception of PfMC-2TM, activation is restricted to a subset of family members, as shown in the box-and-whiskers plot in Fig. 3B. We observed 83.3% of the *PfMC-2TM* family upregulated in the mutant parasites; 46.9% of the *stevor*, 37.2% of the *rif*, and 33.3% *acs* (acyl-CoA synthetase).

We next sought to correlate the presence of the predicted consensus sequence TGCATGCA AP2-exp binding motif in the upstream intergenic regions of the differentially expressed genes in our mutants. Genes containing the motif were compared to our list of differentially expressed genes using the tools available online in plasmodb.org. Surprisingly, only a fraction of these genes contained the motif in their 1500 bp upstream regions (29.7%: 82 out of 276 genes). If the search was restricted to the - 1000 bp, only 25% of the genes had the motif upstream (69 genes out of 276).

**Figure 4:**
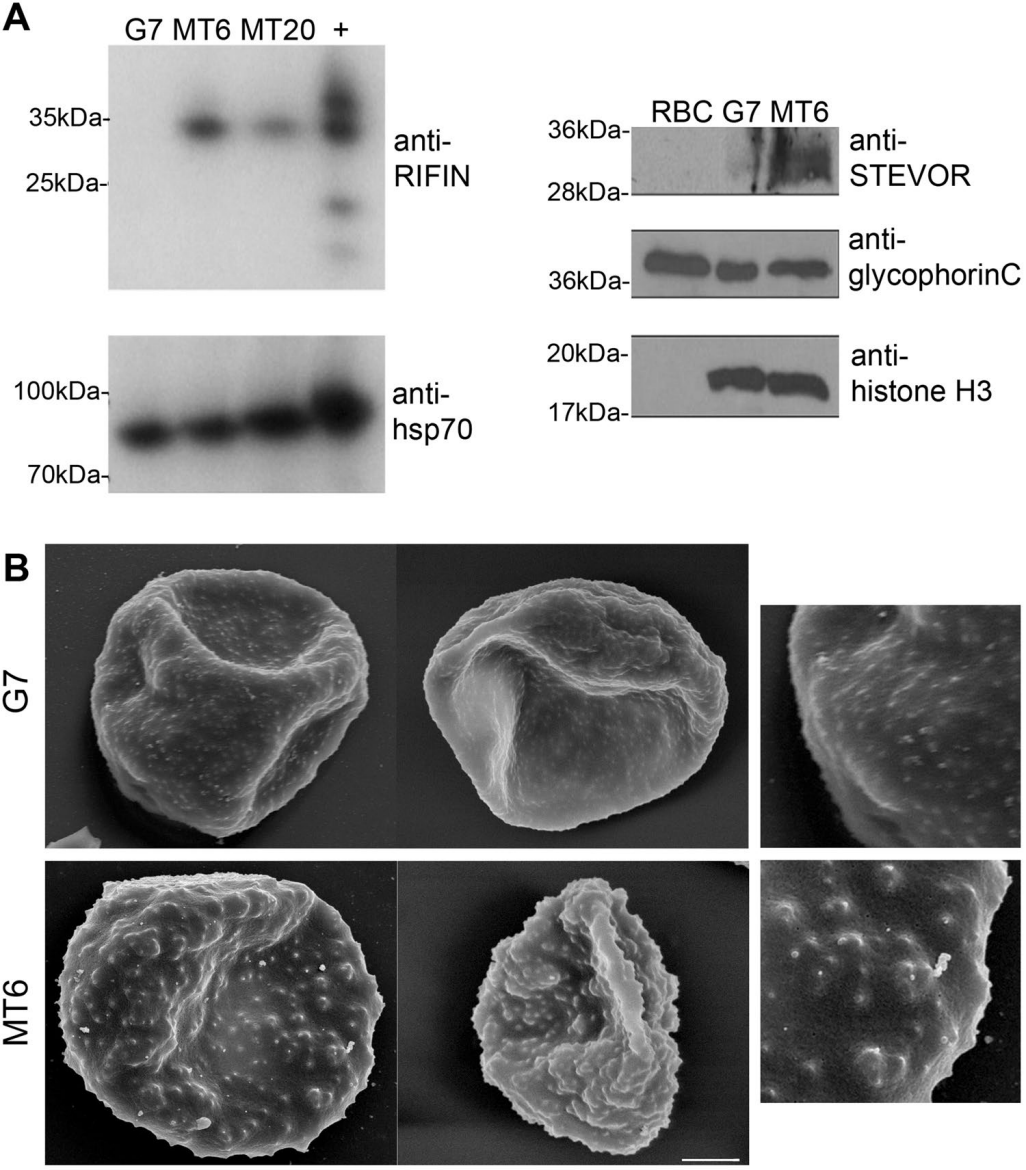
AP2-exp mutant causes upregulation of surface protein expression and morphological changes in the host cell. (**A**) Western blot using antibodies against the conserved C-terminus of RIFINs on extracts of G7, MT6, MT20, and a positive control, FCR3S1.2 (+). Loading control, anti-hsp70. (**B**) Western blot using anti-STEVOR on uninfected RBC (RBC), G7 and MT6. Loading controls: anti-glycophorin (**c**) and histone H3. Cropped bands are shown and full-length membranes are available in Supplementary Figure S6. (**c**) Scanning electron microscopy of RBCs infected with G7 and MT6. Scale bar, 1 μm.

The upregulation of *rif* and *stevor* gene members was extended to the protein levels, as shown by the western blots using antibodies directed against the conserved C-terminus of RIFINs and the conserved N-terminus of STEVORs (Fig. 4A). In both cases, significantly higher protein levels were observed when compared to wild-type expression levels. Taken together, these results point to a role of AP2-exp regulating the expression of clonally-variant surface antigens in *P. falciparum*. Moreover, we hypothesized that such an upregulation of exported proteins might cause morphological changes on the surface of the infected red blood cell. By means of scanning electron microscopy, we observed indeed that the mutant parasites had a rougher, more heterogeneous surface, with apparently larger knobs, when compared to the wild-type parasites (Fig. 4B).

## Discussion

The discovery of the plant-related ApiAP2 DNA-binding family of putative transcription factors opened up new avenues into research of the molecular determinants of gene regulation and key events in malaria parasite development at various life cycle stages^39,40^. It has been assumed that the DNA-binding domains determine the specific interactions with genome loci and in that way regulate genes involved in specific developmental processes during the complex life cycle of *Plasmodium*. In this work we investigated a member of the AP2 family that has been predicted to contribute to the control of one of the most important parasite virulence gene families, termed *var* ^19^, though the motif recognized by this AP2 is the most frequently found octamer in Apicomplexan genomes^41,42^. The predicted consensus sequence TGCATGCA recognized by AP2-exp is found in the intergenic regions (−1500 bp) of 1485 predicted gene targets from plasmodb.org, while it is found 10,068 times in the whole *P. falciparum* genome (browsing the plasmoDB genome for the YRCATGYR pattern).

Firstly, we found that unlike its *P. berghei* orthologue AP2-Sp^24^, sharing 92% identity in the DNA-binding domain, AP2-exp is expressed in the IEC of *P. falciparum*, in late stages. It is interesting how different can the function of the same protein vary between species. It is possible that AP2-exp also plays the role in development of sporozoites, since its transcript is detected in *P. falciparum* sporozoites^43^ but on the other hand, AP2-Sp does not grossly impair survival of the parasites in the murine host^24,30^. It is clear that the more divergent portion of the proteins, the C-terminus, provides distinct functions in the different parasites.

Secondly, only a few genes were differentially regulated when AP2-exp was disrupted, truncating the protein just after the DNA-binding domain. These genes mostly belong to exported multigene families. Given that we could never obtain clean gene knockout parasites by the classical approach (now, the Cas9/CRISPR system might facilitate this task^44^), our data indicates a role for the C-terminus of AP2-exp in regulating exported proteins in *P. falciparum* while the AP2 DNA-binding domain itself plays an essential role which is retained in our truncated mutants. Unfortunately, we could not obtain enough enriched immunoprecipitated DNA from ChIP experiments for a significant genome-wide occupancy analysis of AP2-exp. Tagging the gene using Cas9/CRISPR could be envisaged and ChIP, using ChIP-grade commercial antibodies, could solve this technical issue in the future.

It is known that multigene families in *P. falciparum* are clonally variant and cloned lines can differ in the expression profiles of their members providing a bet-hedging strategy for parasite survival in the host^45^. Our RNA-seq analysis of parasites expressing a truncated AP2-exp revealed transcriptional changes going beyond the observed variegated transcription of subtelomeric gene families (Supplementary Figure S2).

A report from *A. thaliana* indicates the binding of an AP2 member to many regions in the genome but only a few genes have their transcriptional status modified when the AP2 protein is knocked out; even introns were found to be bound by this protein^46^. Likewise, a recent report on *P. berghei* shows that AP2-O binds to around 1000 5′ UTRs and only a fraction of these genes are regulated in the ookinete stage^28^. More recently, it has been shown that *P. falciparum* AP2 family is subjected to protein acetylation and this modification can alter DNA-binding^47^, thus adding another layer of complexity on transcriptional regulation in malaria parasites. We have used our anti AP2-exp antibodies to explore the binding sites for this AP2 member using ChIP-seq. The antibodies did result in a general genome-wide distribution of AP2-exp although with no statistically significant enrichment at genes that showed transcriptional changes. We assume that our antibody is not ChiP-grade or that protein interactions with AP2-exp mask the epitopes recognized by our antibody, as additionally it did not detect the protein in immunofluorescence assays.

Most importantly, we demonstrate a role for the non-DNA-binding domain of an ApiAP2 member in the regulation of expression of some clonally-variant gene families such as *rif*, *stevor* and *Pfmc-2TM*. For those gene families, no regulatory molecule has been identified so far. How the C-terminus of AP2-exp contributes to the regulation of only a subset of genes remains unknown. Since these genes are known to be expressed at different time points during the parasite life cycle, it is possible that RNA-seq analysis of more time points during the IEC will reveal deregulation of other members of these gene families.

Our RNA-seq data revealed differential expression of a few *var* genes at the trophozoite stage, possibly due to a lack of proper general repression mechanisms of subtelomeric genes in the mutant AP2-exp parasites. Our data point to a particular role of AP2-exp for clonally variant gene families that depend on the heterochromatin environment to express only one or few members of the corresponding gene families (variegated gene expression). In fact, two hypothesis might explain the gene expression deregulation observed in our AP2-exp mutants: 1) either the lack of the C-terminal domain impairs the downstream functions of AP2-exp, abolishing its function, supporting its role as a transcriptional repressor or 2) the overexpressed truncated AP2-domain could be binding to more accessible target sites, or even “nonspecific” sites, increasing activation of these genes, supporting a transcriptional activator role for AP2-exp. We support the first hypothesis, since we could not observe increased enrichment of the truncated AP2-exp in ChIP experiments on our mutants, compared to the wild-type signals, and we did not find enriched motifs in the 5′ UTRs of the differentially expressed genes in the mutant parasites that could point to possible nonspecific sites.

Our work suggests that specific chromatin factors could be recruited by AP2-exp to promote gene repression. Proteomic analysis on the protein complexes harboring the AP2-exp proteins may further advance our mechanistic insight. Genomic loci-specific pull downs are needed for this and new techniques such as deadCas9/CRISPR are emerging as a new approach to tackle this type of questions in *Plasmodium* in the future^44,48^. It is important to note that no defined domain could be found in the adjacent regions of the AP2 domain that would predict interactions with other proteins or DNA (data available in plasmodb.org). Clearly, more functional studies are needed to elucidate their biological role in the gene regulation process.

## Conclusions

We have characterized a member of the AP2 family that adds a new dimension to the biological features so far associated to this related group of DNA-binding proteins. RNA-seq analysis of mutant parasites expressing only a short truncated AP2-exp (including the DNA-binding domain) reveal that many members of clonally variant gene families are derepressed, showing a specific regulatory function for the non-DNA-binding region of AP2-exp. These deregulated genes are mostly surface-exported proteins and the upregulation of two of these families, *rif* and *stevor* are observed up to the protein levels. We also observed differences at the surface of the RBCs infected by the mutants. So far, it remains unclear how AP2-exp targets specific subsets of subtelomeric gene families. Further studies may provide mechanistic insight of how AP2-exp is recruited to the genome and how it does exert regulatory functions to a subset of subtelomeric genes in *P. falciparum*. Taken together, our work points to the existence of complex regulatory networks with a central position of AP2-exp in connecting the expression of different subtelomeric virulence gene families.

## Methods

### Parasite culture

*Plasmodium falciparum* 3D7 and derived strains were cultivated in human O + red blood cells in RPMI-1640 medium supplemented with Albumax II, hypoxanthine and gentamycin in 3% CO_2_ and 5% O_2_ at 37 °C. Parasite synchronization (6 h window) was periodically done by gelatin flotation and sorbitol treatment as described^49^. Transfections were done by electroporation of ring stages using 100 µg plasmid purified using NucleoBond Xtra kit (Macherey Nagel) as described^49,50^. Positive drug selection was done using 2.66 nM WR99210 for pCC1 transfection.

### Plasmid constructs

Homology boxes were cloned into pCC1 plasmid^51^ by PCR amplification of the desired regions from 3D7 genomic DNA using specific primers flanked by appropriate restriction sites (list of primers, see Supplementary Table 1). Ligation was done using T4 DNA ligase (NEB) and transformed into XL10-Gold ultracompetent cells (Agilent).

For protein expression, we designed primers to amplify the N-terminal region of AP2-exp encompassing the AP2 DNA-binding domain into the pGEX-B vector (Pharmacia/GE Healthcare/Life Technologies). PCR products were purified and ligated in the vector using T4 DNA ligase and the ligation product, transformed into chemically-competent DH5α *E. coli* cells. Sequencing of recombinant clones was done to confirm the correct ligation of the gene in frame with the glutathione S-transferase in the pGEX plasmid and BL21(DE3)RIL *E. coli* was transformed to obtain clones for protein expression. Protein expression was done inducing overexpression for 6 hours at 37 °C after addition of isopropyl β-D-thiogalactopyranoside (IPTG, 0.1 mM). Protein was purified on Glutathione Sepharose 4B columns and sent to Genscript for immunization.

### Antibody and protein methods

Antibodies anti-AP2-exp were obtained from rats immunized with the recombinant GST-tagged N-terminal region of the AP2 protein using standard protocols by Genscript. As controls, anti-PfAldolase and anti-core human H3 were used (Abcam). For western blotting, cytosolic and nuclear extracts of saponin-lysed parasites (as described above) were obtained after stroking parasites in a Potter homogeneizer, diluted in SDS-PAGE loading buffer (Bio-Rad) for a final 1x concentration and loaded on 4–12% Criterion gels (Bio-Rad). Transfer was done onto nitrocellulose membranes using iBlot system (Life) and blocking and antibody incubations in PBS 0.1% tween-20 (PBST) containing 5% milk. Peroxidase-conjugated secondary antibodies were from GE Healthcare and luminescent signals after incubation with femto ECL reagent (Thermo Scientific) acquired in a GelDoc system (Bio-Rad).

For the anti-STEVOR western blots, carbonate-insoluble pellet fraction of Gelofusine-enriched late stage *P. falciparum* iRBC and uninfected RBC extracts were used. Briefly, the cells were first lysed in 10 volumes of 10 mM Tris-HCl, pH 7.4, containing protease inhibitors (Roche) and 1 mM EDTA, for 30 min on ice. The lysate was centrifuged at 20000x g for 30 min at 4 °C. Proteins from the pellet were then extracted for 30 min with 10 volumes of 0.1 M sodium carbonate pH 11.5 with protease inhibitors and 1 mM EDTA, at 4 °C and centrifuged as previously described^52^. Cells (10^6^) and parasites (10^5^) equivalents of the carbonate-insoluble fraction were loaded on SDS-PAGE for immunoblotting. All antibodies used were diluted in PBST with 5% milk. Rabbit polyclonal anti-STEVOR (anti-S1) at 1:3000 dilution, rabbit anti-glycophorin C (Abcam) and anti-H3 (Abcam) both at 1:1000 dilution were probed with the nitrocellulose membrane for 1 h at room temperature (RT) or 4 °C overnight. Four washes of 10 min each with 1X PBST were performed. Secondary anti-rabbit HRP (GE Healthcare) was used at 1:5000 dilution for 1 h at RT and the blots were then washed as above. Subsequently, chemiluminescent substrate (ECL) (Thermo Scientific) was used for detection.

### Scanning electron microscopy

After gelatin flotation, *P. falciparum*-infected erythrocytes were resuspended in PBS. A drop of the cell suspension was allowed to settle on a glass coverslip coated with 0.1% poly-L-lysine (Sigma Aldrich). After 10 min, the unattached cells were removed. The infected RBC were fixed in 2.5% glutaraldehyde in PBS buffer overnight at 4 °C. After three washing steps in PBS, the sample was post-fixed for 1 hour in 1% osmium tetroxide (Electron Microscopy Sciences). After three washing steps in water, the sample was serially dehydrated with 25, 50, 75, 95 and 100% ethanol, followed by critical point drying. The coverslip was sputter-coated with a thin layer of 10 nm gold. Then, samples were analyzed with a JSM-6700F electron microscope (Jeol) using secondary electrons at 5 kV.

### RNA extraction, cDNA synthesis and quantitative PCR

Total RNA from saponin-lysed parasites was extracted by Trizol (Life), DNAse-treated by Turbo DNAse kit (Life) and cDNA was prepared from 500–1000 ng total RNA using SuperScript II RT kit (Life) and random hexamers. Quantitative PCR was performed using specific primers for single *var* genes (Salanti^51^, Supplementary Table S1 or specific primers for selected target genes with Power SyBr Green PCR Master Mix (Thermo Scientific). Standard curves were prepared from 49-fold dilutions of genomic DNA from 3D7 parasites and quantities were normalized to either seryl-tRNA synthetase or the geometric means of seryl-tRNA synthetase (PF3D7_0717700), fructose-biphosphate aldolase (PF3D7_1444800) and inositol 5-phosphatase (PF3D7_0802500) for relative copy number determination.

### Transcriptomic analysis (RNA-seq)

RNA was obtained from saponin-lysed parasites parasites synchronized at 30 hpi using miRNEasy kit (Qiagen), followed by polyA-enrichment using Dynabeads Direct mRNA isolation kit (Life Technologies). RNA was fragmented using Ambion fragmentation kit (Thermo Scientific) and libraries were prepared as described using TruSeq Small RNA kit^53^ for Illumina sequencing in a HiSeq 2500. Reads were either 50 bp- or 100 bp-long.

### Next generation sequencing and data analysis

After initial quality control using FastQC software (http://www.bioinformatics.babraham.ac.uk/projects/fastqc/), reads were mapped to the *Plasmodium falciparum* 3D7 genome (v3, PlasmoDB) using the BWA MEM software^54^ at default settings. The edgeR library^55^ in the R statistical environment was used to normalize, explore, and estimate significantly differentially expressed (DEX) genes from the RNA expression data.

### Availability of supporting data

The datasets supporting the results herein are available in the EMBL-EBI European Nucleotide Archive, accession number: PRJEB20833 (http://www.ebi.ac.uk/ena).

## Electronic supplementary material

supplementaryInfo

SupplementaryTable2
