# Supplementary material for: An ApiAP2 member regulates expression of clonally variant genes of the human malaria parasite Plasmodium falciparum
Source: Sci Rep. 2017 Oct 25;7:14042. doi: 10.1038/s41598-017-12578-y (PMC5656681; doi:10.1038/s41598-017-12578-y)

Supplementary Information

"An ApiAP2 member regulates expression of clonally variant genes of the human malaria parasite *Plasmodium falciparum* "

Rafael M. Martins, Cameron R. Macpherson, Aurélie Claes, Christine Scheidig-Benatar, Hiroshi Sakamoto, Xue Yan Yam, Peter Preiser, Suchi Goel, Mats Wahlgren, Odile Sismeiro, Jean-Yves Coppée and Artur Scherf.

Description of the supplementary files:

Supplementary table S1: List of oligonucleotides used in the study.

Supplementary table S2: List of differentially-expressed genes in the mutant AP2-exp (ST2.xls file)

Supplementary figure S1: Localization of differentially-expressed genes in the mutant AP2-exp clones on *P. falciparum* chromosomes.

Supplementary figure S2: Venn diagram of differentially-expressed genes in AP2-exp mutants versus genes reported to contain the predicted binding motif of AP2-exp in their 5'UTR.

Supplementary figure S3: Full-length membrane of western-blot of anti-AP2-exp (related to figure 1).

Supplementary figure S4: Full-length agarose gel of diagnostic PCR for wild-type and mutant parasites (related to figure 1)

Supplementary figure S5: Full-length membrane of western-blot of loading controls anti-Pfaldolase and anti-histone H3 (related to figure 2).

Supplementary figure S6: Full-length membrane of western-blot of anti-STEVOR and loading controls anti-glycophorin C and anti-histone H3 (related to figure 4).

Supplementary table S1: List of oligonucleotides used in the study

#integration-checking oligos

1: PF14-5’: GAAAAATACAAAATAAGCAGTCTA

2: PF14-3’: GGTTCATATCTATGGTTTGTGA

3: PTKATG5’: CTTTAAATTCATGCAAAAATTTAC

4: PTKTAA3’: AACTATGCGGCATCAGAGCAG

#cloning oligos for pCC1 plasmid

Box1Fwd: atg ccgcgg ATGGAAGATAACAATATGAATAATGAACAAAAATTTAATCCTAATATGTT

Box1Rev: agg actagt TCAAGATTTAATTAAATCACTTACACCAGTATTCACATTCC

Box2Fwd: cct gaattc GGTTTACATAATAAAGATGTAGAAAACTTGATGAATGTGTTATTTAGGC

Box2Rev: ttg cctagg ATTGGAATCTCCTAAGTTCCTTCCACTATTTCCATTAGAATTAT

#cloning oligos for recombinant protein expression in pGEX

PF14_0633NF1: GCGGGATCC ATGGAAGATAACAATATGAATAATGAACAAAAATTTAATC

PF14_0633NR1: CGCGAATTC TTAATGATTATTCAAAGAGTTAAACATATTATTTCTTTCG

#qPCR oligos for homology boxes quantification

ko14b1f1: GTTAGCCAAGAACCAGCAG

ko14b1r1: CAACTAACACCAGGATAACCAG

ko14b2f1: CGATTCAAATAATAACAACC

ko14b2r1: TTG TCT CAA TTG ATT TAT CC

#qPCR oligos for normalization

Gene ID plasmoDB	annotation	fwd oligo	rev oligo
PF3D7_0717700	seryl-tRNA synthetase	AAGTAGCAGGTCATCGTGGTT	TTCGGCACATTCTTCCATAA
PF3D7_0802500	inositol 5-phosphatase	GACATAAGTTTAGTAGGTCG	TTCTGACTCCACATCATTTG
PF3D7_1444800	fructose-biphosphate aldolase	TGTACCACCAGCCTTACCAG	TTCCTTGCCATGTGTTCAAT

#qPCR oligos for var genes (based on Salanti 2003)

GeneID plasmoDB	var subgroup (Lavstsen 2003)	fwd oligo	rev oligo
PF3D7_1300300	A	CAC AGG TAT GGG AAG CAA TG	CCA TAC AGC CGT GAC TGT TC
PF3D7_0425800	A	AAA CAC GTT GAA TGG CGA TA	GAC GCC GAG GAG GTA AAT AG
PF3D7_0100300	A	TCA TTA TGG GAA GCA CGA TT	TGA TTT CTA CCA TCG CAA GG
PF3D7_0800200	A	GGT GTC AAG GCA GCT AAT GA	TAT GTC CTG CGC TAT TTT GC
PF3D7_0400400	A	ATA TGG GAA GGG ATG CTC TG	TGA ACC ATC GAA GGA ATT GA
PF3D7_1100200	A	GAC GGC TAC CAC AGA GAC AA	CGT CAT CAT CGT CTT CGT TT
PF3D7_0600400	A	CGT AAA ACA TGG TGG GAT GA	GGC CCA TTC AGT TAA CCA TC
PF3D7_1150400	A	TGC TGA AGA CCA AAT TGA GC	TTG TTG TGG TGG TTG TTG TG
PF3D7_0937600	A	TGA CCAAGACGAAGTATGGAA	TTGATCTCTGTTCGCTGTCC
PF3D7_0632800	B	GAC AAA TAC GGC GAC TAC GA	TGT TTCACC CCA TTC TTC AA
PF3D7_0800100	B	GTC GTG GAA AAA CGA AAG GT	TAT CTA TCC AGG GCC CAA AG
PF3D7_1000100	B	GAC GAG GAG TCG GAA AAG AC	TGG ACA GGC TTG TTT GAG AG
PF3D7_1041300	B	GTG CAC CAA AAG AAG CTC AA	ACA AAA CTC CTC TGC CCA TT
PF3D7_1100100	B	GAG GCT TAT GGG AAA CCA GA	AGG CAG TCT TTG GCA TCT TT
PF3D7_1300100	B	ACA AAG GAA CGT CCA TCT CC	GCC AAT ACT CCA CAT GAT CG
PF3D7_1373500	B	CGG AAT TAG TTG CCT TCA CA	CAT TGG CCA CCA AGT GTA TC
PF3D7_0100100	B	TGC GCT GAT AAC TCA CAA CA	AGG GGT TCA TCG TCA TCT TC
PF3D7_0115700	B	AAC CCC CAA TAC CAT TAC GA	TTC CCC ACT CAT GTA ACC AA
PF3D7_0200100	B	ATG TGC GCT ACA AGA AGC TG	TTG ATC TCC CCA TTC AGT CA
PF3D7_0223500	B	CAA TTT TGG GTG TGG AAT CA	CAC TGG CCA CCA AGT GTA TC
PF3D7_0324900	B	CAA TCT GCG GCA ATA GAG AC	CCA CTG TTG AGG GGT TTT CT
PF3D7_0400100	B	GAC GAC GAT GAA GAC GAA GA	AGA TCT CCG CAT TTC CAA TC
PF3D7_0426000	B	TGA CGA CTC CTC AGA CGA AG	CTC CAC TGA CGG ATC TGT TG
PF3D7_0900100	B	TGC AAA CCA CCA GAA GAA AG	GTT CTC CGT GTT GTC CTC CT
PF3D7_1200100	B	CGG AGG AGG AAA AAC AAG AG	TGC CGT ATT TGA GAC CAC AT
PF3D7_1219300	B	GAC GCC TGC ACT CTC AAA TA	TTG GAG AGC ACC ACC ATT TA
PF3D7_1255200	B	GGC ACG AAG TTT TGC AGA TA	TTT GTG CGT CTT TCT TCG TC
PF3D7_0500100	B	GAA GCT GGT GGT ACT GAC GA	TAT TTT CCC ACC AGG AGG AG
PF3D7_0937800	B	CAC ACG TGG ACC TCA AGA AC	AAA ACC GAT GCC AAT ACT CC
PF3D7_0733000	B	TGA CGA CGA TAA ATG GGA AA	TTC TTT TGG AGC AGG GAG TT
PF3D7_0632500	B/A	ATG TGT GCG AGA TGA AG	TGC CTT CTA GGT GGC ATA CA
PF3D7_1200400	B/A	TCG ATT ATG TGC CGC AGT AT	TTC CCG TAC AAT CGT ATC CA
PF3D7_0800300	B/A	TTT GGG ATG ACA CCA AGA AA	GTC GCT TGA TGA AGG AGT CA
PF3D7_0600200	B/A	TGG AAA GAA CAT GGA CCT GA	TTC CTC GAG GGA AGA ATC AC
PF3D7_0413100	B/C	ACT TTC TGG TGG GGA ATC AG	TTC ACC GCC ACT TCA TTC AG

PF3D7_0712800	B/C	ACG TGG TGG AGA CGT AAA CA	CCT TTG TTG TTG CCA CTT TG
PF3D7_0712400	B/C	GCG ACG CTC AAA AAC ATT TA	TCA TCC AAC GCA ATC TTT GT
PF3D7_0712300	B/C	GGT GGA GGT AGT CCA CAG GA	CAG CTA TTT CCC CAC CAG AA
PF3D7_0809100	B/C	TGC AAG GGT GCT AAT GGT AA	CCT GCA TTT TGA CAT TCG TC
PF3D7_0808700	B/C	TTT GTC CGG AAG ACG ATA CA	ATC TGG GGC AGA ATT ACC AC
PF3D7_1240300	B/C	AGC AAA ATC CGA AGC AGA AT	CCC ACA GAT CTT TTC CTC GT
PF3D7_1240400	B/C	GGTGATACCACGATGATTGA	CACCAGGTTTTACGTTGTTC
PF3D7_0421100	B/C	ACCACATCTAGTAGTGCCA	AGCAGAACTCTCGATAAACC
PF3D7_0421300	B/C	TGACCACAAAAGTGTGAAAC	CCCATATCTTTTGCATGCTG
PF3D7_1240600	C	CAT CCA TTA CGC AGG ATA CG	AAA TAG GGT GGG CGT AAC AC
PF3D7_0412700	C	AAGCATCTGAGGATCATATAAA	CATTTCAATTTGTACGTTAGTGG
PF3D7_0617400	C	ATT TGT CGC ACA TGA AGG AA	AAC TTC GTG CCA ATG CTG TA
PF3D7_0412400	C	ACC GCC CCA TCT AGT GAT AG	CAC TTG GTG ATG TGG TGT CA
PF3D7_0420700	C	AGA GGG TTA TGG GAA TGC AG	GCA TTC TTT GGC AAT TCC TT
PF3D7_0712000	C	GTT GAG TCT GCG GCA ATA GA	CTG GGG TTT GTT CAA CAC TG
PF3D7_0712600	C	CGT GGT AGT GAA GCA CCA TC	CCC ACC TTC TTG TGG TTT CT
PF3D7_0711700	C	CAA TTT TTC CGA CGC TTG TA	CAC ATA TAG CGC CGT CCT TA
PF3D7_0712900	C	CAC ACA TGT CCA CCA CAA GA	ACC CTT CTG TGG TGT CTT CC
PF3D7_0808600	C	CCT AAA AAG GAC GCA GAA GG	CCA GCA ACA CTA CCA CCA GT
PF3D7_1240900	C	AAA GCC ACT AGC GAG GGT AA	TGT TTT TGC CCA CTC CTG TA
PF3D7_0533100	var1	AAG AAA GTG CCA CAA CAT GC	GTT CGT ACG CCT GTC GTT TA
PF3D7_1200600	var2	CAC GAC ATT AAC AAT ACA TGC AGA	CAT TGC ATT CAC AGA CAT TGG

Supplementary table S2: List of differentially-expressed genes in the mutant AP2-exp (ST2.xls file, separate file)

Supplementary figure S1: Localization of differentially-expressed genes in the mutant AP2-exp clones on *P. falciparum* chromosomes

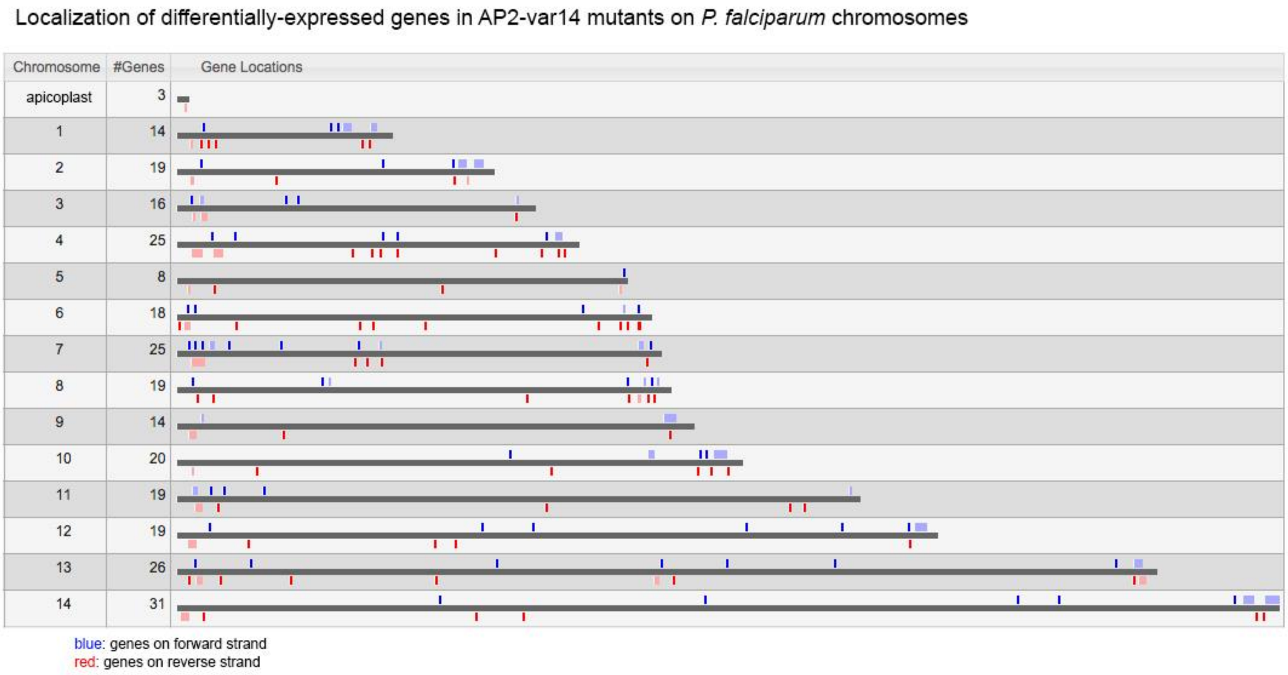

Supplementary figure S2: Venn diagram of differentially-expressed genes in AP2-exp mutants versus genes belonging to the Rovira-Graells N. et al. (reference 43) variantome of 3D7 parasites.

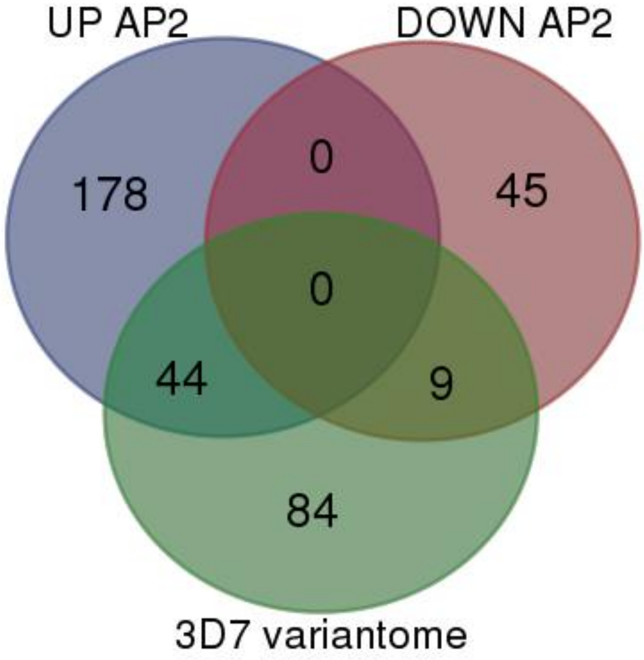

Supplementary figure 3: original full-length membranes of figure 1B : 2 different exposures of the western-blot using anti-AP2-exp. On the right, molecular weight markers in kDa, C, cytoplasmic extract, N, nuclear extract.

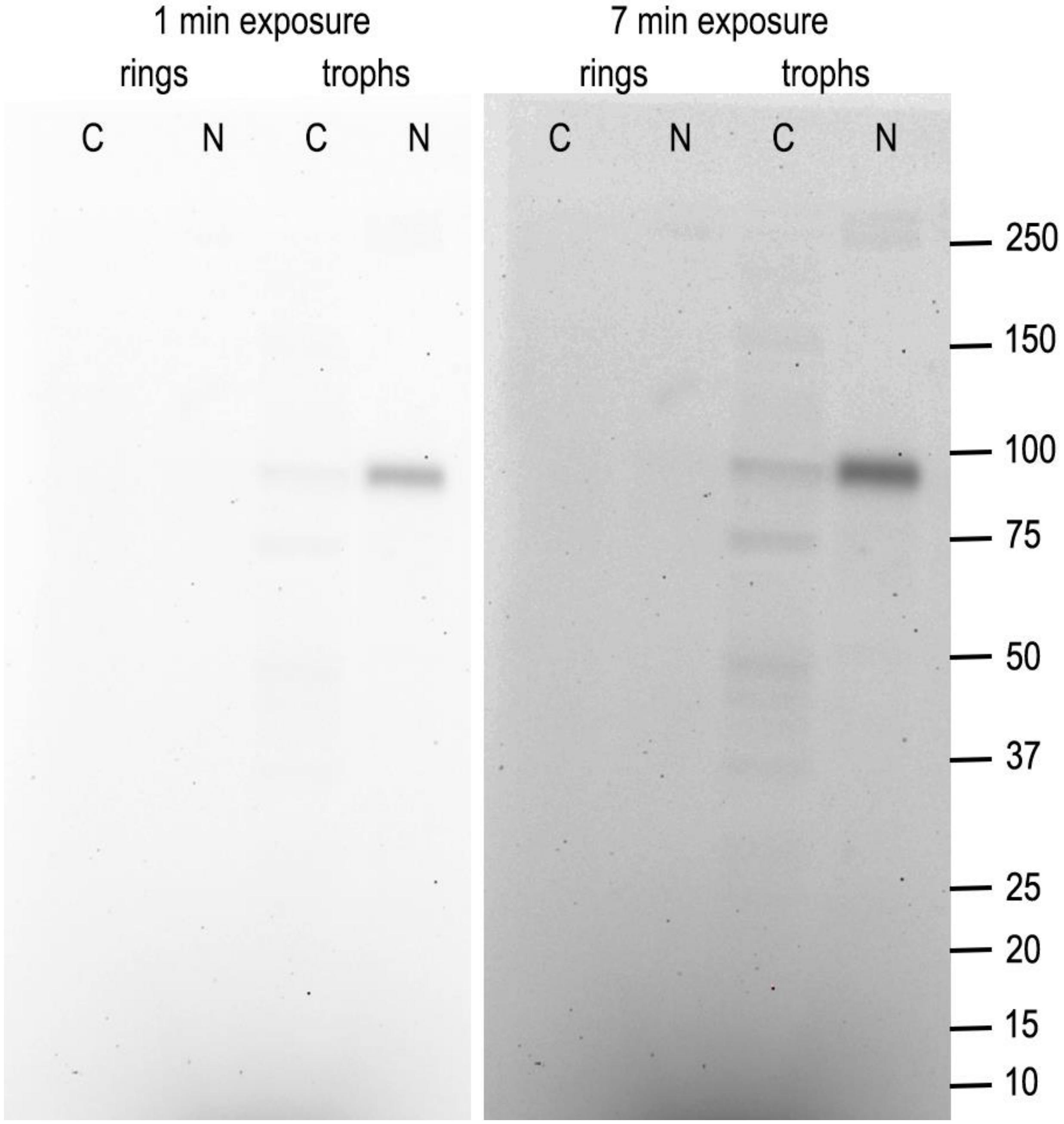

Supplementary figure 4: original full-length agarose gel of figure 1C. Left gel, wild-type G7 PCR reactions and right gel, mutant 6 ones. Mk: molecular weight markers, indicated on the left in kbp. 1+2: locus PCR, as shown in figure 1C; HR1: integration of HR1; HR2: integration of HR2; 3+4: episome detection, as shown in figure 1C.

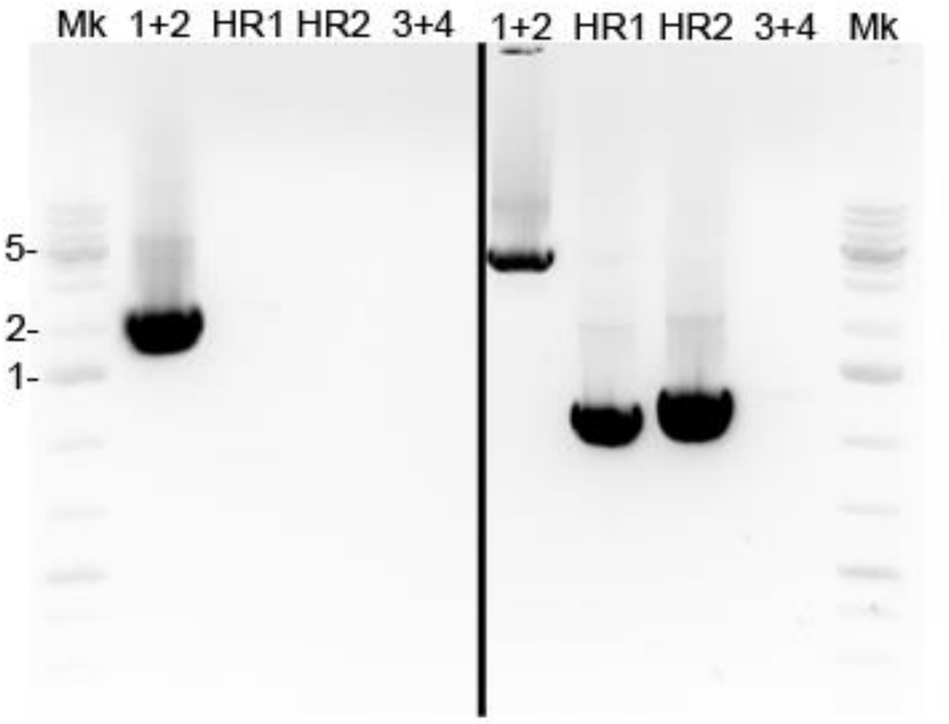

Supplementary figure 5: original full-length membrane of figure 2D : anti-Pfaldolase (indicated by black arrow) and anti-histone H3 (indicated by grey arrow). On the left, a crop of the molecular weight markers on the scanned membrane.

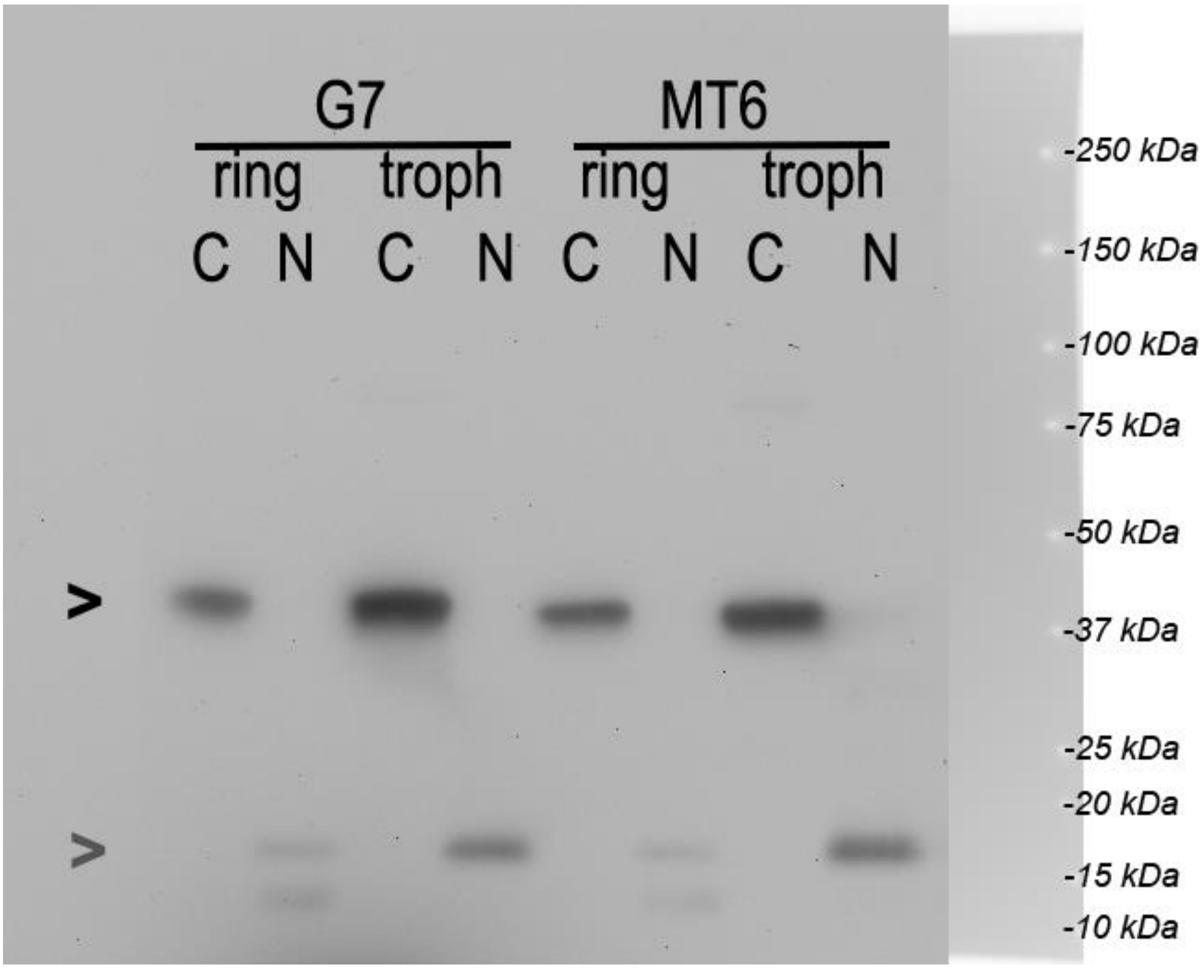

Supplementary figure 6: original full-length membranes of figure 4A : loading controls anti-glycophorin C and anti-histone H3 and anti-STEVOR, as indicated below each membrane. Gel at the bottom is anti-histone H3 incubated on the anti-glycophorin C membrane.

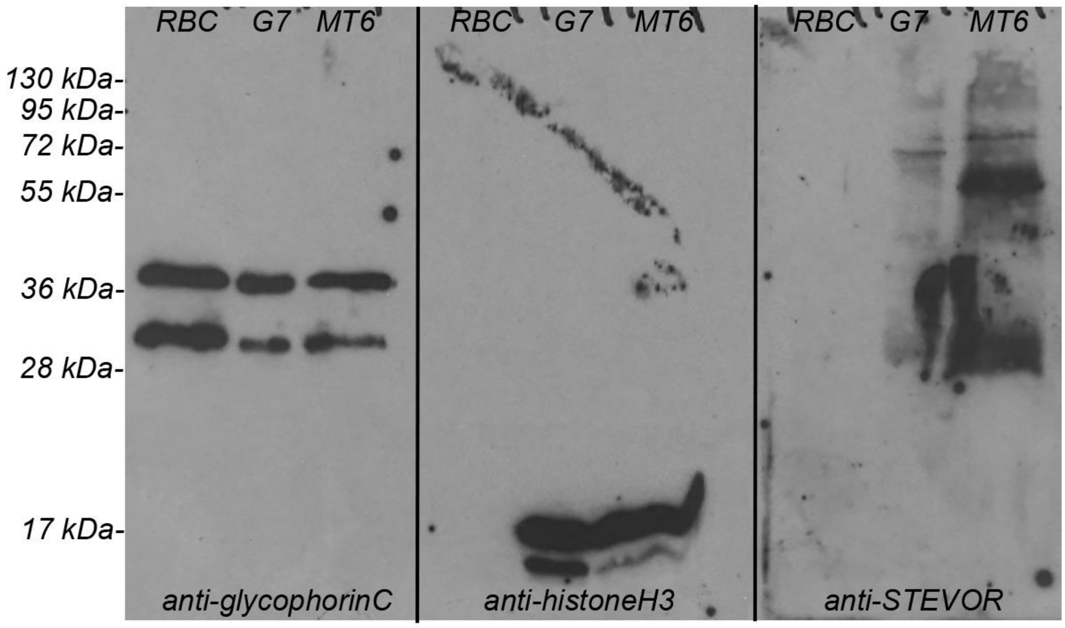

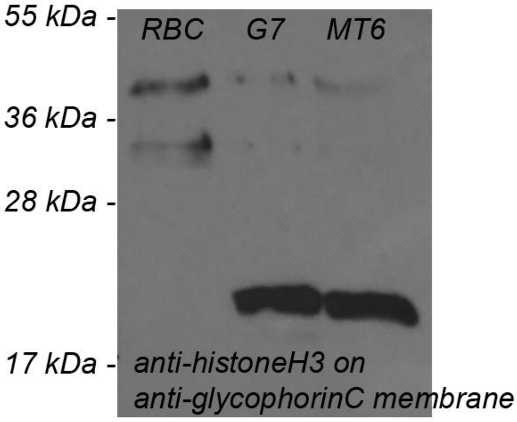

Supplement: Supplementary file 1 — supplementaryInfo [file 41598_2017_12578_MOESM1_ESM.pdf]
